# Predictive Significance of Combined Plasmatic Detection of BRAF Mutations and S100B Tumor Marker in Early‐Stage Malignant Melanoma

**DOI:** 10.1002/cam4.70313

**Published:** 2024-10-10

**Authors:** Jiri Polivka, Mohamed A. Gouda, Mahyar Sharif, Martin Pesta, Helen Huang, Inka Treskova, Vlastimil Woznica, Jindra Windrichova, Katerina Houfkova, Radek Kucera, Tomas Fikrle, Jan Ricar, Kristyna Pivovarcikova, Ondrej Topolcan, Filip Janku

**Affiliations:** ^1^ Department of Histology and Embryology, Faculty of Medicine in Pilsen Charles University Pilsen Czech Republic; ^2^ Biomedical Center, Faculty of Medicine in Pilsen Charles University Pilsen Czech Republic; ^3^ Department of Investigational Cancer Therapeutics The University of Texas MD Anderson Cancer Center Houston Texas USA; ^4^ Department of Biology, Faculty of Medicine in Pilsen Charles University Pilsen Czech Republic; ^5^ Department of Plastic Surgery University Hospital Pilsen Pilsen Czech Republic; ^6^ Department of Immunochemical Diagnostics University Hospital Pilsen Pilsen Czech Republic; ^7^ Department of Pharmacology, Faculty of Medicine in Pilsen Charles University Pilsen Czech Republic; ^8^ Department of Dermatovenerology University Hospital Pilsen Pilsen Czech Republic; ^9^ Department of Pathology University Hospital Pilsen Pilsen Czech Republic

**Keywords:** BRAF V600E, ctDNA, ddPCR, melanoma, S100B

## Abstract

**Background:**

Melanoma is the most aggressive skin cancer with ability to recur also after early‐stage tumor surgery. The aim was to identify early‐stage melanoma patients at high risk of recurrence using liquid biopsy, estimating of mutated BRAF ctDNA and the level of tumor marker S100B in plasma.

**Methods:**

Eighty patients were enrolled in the study. BRAF V600E mutation was determined in FFPE tissue and plasma samples using ultrasensitive ddPCR with pre‐amplification. The level of S100B was determined in plasma by immunoassay chemiluminescent method.

**Results:**

The best prediction of melanoma recurrence after surgery was observed in patients with combined high level of S100B (S100B^high^) and ctDNA BRAFV600E (BRAF^mut^) in preoperative (57.1% vs. 12.5%, *p* = 0.025) as well as postoperative blood samples (83.3% vs. 14.3%, resp., *p* = 0.001) in comparison with low S100B and BRAF wild‐type. Similarly, patients with preoperative and postoperative S100B^high^ and BRAF^mut^ experienced worse prognosis (DFI *p* = 0.05, OS *p* = 0.131 and DFI *p* = 0.001, OS = 0.001, resp.).

**Conclusion:**

We observed the benefit of the estimation of combination of S100B and ctDNA BRAF^mut^ in peripheral blood for identification of patients at high risk of recurrence and unfavorable prognosis.

**Significance:**

There is still no general consensus on molecular markers for deciding the appropriateness of adjuvant treatment of early‐stage melanoma. We have shown for the first time that the combined determination of the ctDNA BRAF^mut^ oncogene (liquid biopsy) and the high level of tumor marker S100B in pre‐ and postoperative plasma samples can identify patients with the worst prognosis and the highest risk of tumor recurrence. Therefore, modern adjuvant therapy would be appropriate for these patients with resectable melanoma, regardless of disease stage.

## 1 Introduction

Melanoma represents the most aggressive skin cancer with unfavorable prognosis and high mortality, especially in metastatic disease [1]. Melanoma in early stages undergoing surgery might also experience recurrence within the two years after tumor resection in 13% of cases [2]. Despite the recent advances with targeted therapies and immunotherapies in melanoma adjuvant treatment, and even in neoadjuvant settings [3], high costs and the possible serious side effects represent limitations of that approaches [4, 5]. Therefore, better identification of patients at high risk of recurrence after melanoma surgery who can benefit most from such therapies is needed.

For several decades, the tumor marker S100B has been used to determine the prognosis of patients with advanced melanoma with ambiguous benefit [6, 7, 8]. The S100 proteins represent a family of low‐molecular‐weight calcium‐binding proteins that are well known for their vital role in cell differentiation, apoptosis, proliferation, calcium homeostasis, or inflammation [9]. S100 proteins are also significantly important factors in developing various diseases and progression in different cancer types including melanoma [9, 10]. Among the S100 family members, the S100A1, S100A2, S100A4, S100A6, S100A7, S100A8, S100A9, S100A10, S100A11, S100A13, and S100B dysregulation has been reported in different disease stages [11, 12]. S100 expression level is also prognostic marker in melanoma and can serve as an indicator for treatment efficacy [5, 11].

In addition to S100, the liquid biopsy approach is gaining interest in determining prognosis and predicting treatment, specifically in patients with malignant melanoma through the detection of BRAF mutations in circulating tumor DNA (ctDNA) or the evaluation of circulating tumor cells (CTC) [13, 14, 15]. Although each of these markers can determine the prognosis of melanoma patients, each of them reflects different features of the process of carcinogenesis. While ctDNA indicates the genetic mutations present in the population of tumor cells from tissue itself [16], CTCs reflect the phenotypically specific population of cells with the ability to migrate from the tumor site into the bloodstream [17]. So the combination of protein and molecular genetic biomarkers could be beneficial in the determination of prognosis in early‐stage melanoma patients.

In this study, we hypothesize that multi‐omics testing, using both ctDNA as well as protein tumor biomarker S100B, may be beneficial as a prognostic biomarker of melanoma recurrence after surgery in early‐stage patients (stages 0—III, 48% in stages 0—IIA). Identification of high‐risk patients in terms of disease recurrence using this approach would allow better identification of patients for whom adjuvant therapy with targeted or immunotherapy may be appropriate.

## 2 Materials and Methods

### 2.1 Patients

Eighty patients with melanoma of stages 0 to III treated at the University Hospital Pilsen were enrolled in the study. The study was approved by the Institutional Review Board and local Ethics Committee of the University Hospital in Pilsen (no. 04112019). Patients provided a signed informed consent form. The melanoma diagnosis and TNM staging was verified by the dermatopathologists according to the American Joint Committee of Cancer, Melanoma of the Skin Staging (eighth edition). Patients with medical records showing another primary tumor were excluded from analysis.

### 2.2 Blood Samples and Plasma Separation

Plasma samples were obtained from patients according to predefined time points (before surgery, after surgery, postoperative day 2, day 3, week 1, week 2, week 3, and then in 6 months in the follow‐up). Two‐step centrifugation of 6 mL of blood, collected into K3EDTA Vacutainer tubes (Greiner Bio‐One, Kremsmünster, Austria), was used for plasma separation (950 relative centrifugal force (RCF) for 10 min at 4°C and then 11,000 RCF for 10 min at 4°C) and plasma was stored at −80°C.

### 2.3 DNA Isolation From Tumor Tissue and Plasma Samples

QIAamp Circulating Nucleic Acid Kit (QIAGEN, Hilden, Germany) was used for the cell‐free DNA (cfDNA) isolation from an average of 3 mL plasma. QIAamp DNA FFPE Tissue Kit (QIAGEN, Hilden, Germany) was used for DNA isolation from corresponding tumor tissue (FFPE blocks).

### 2.4 Detection of BRAFV600E Mutation by Droplet Digital PCR

**FIGURE 1:**
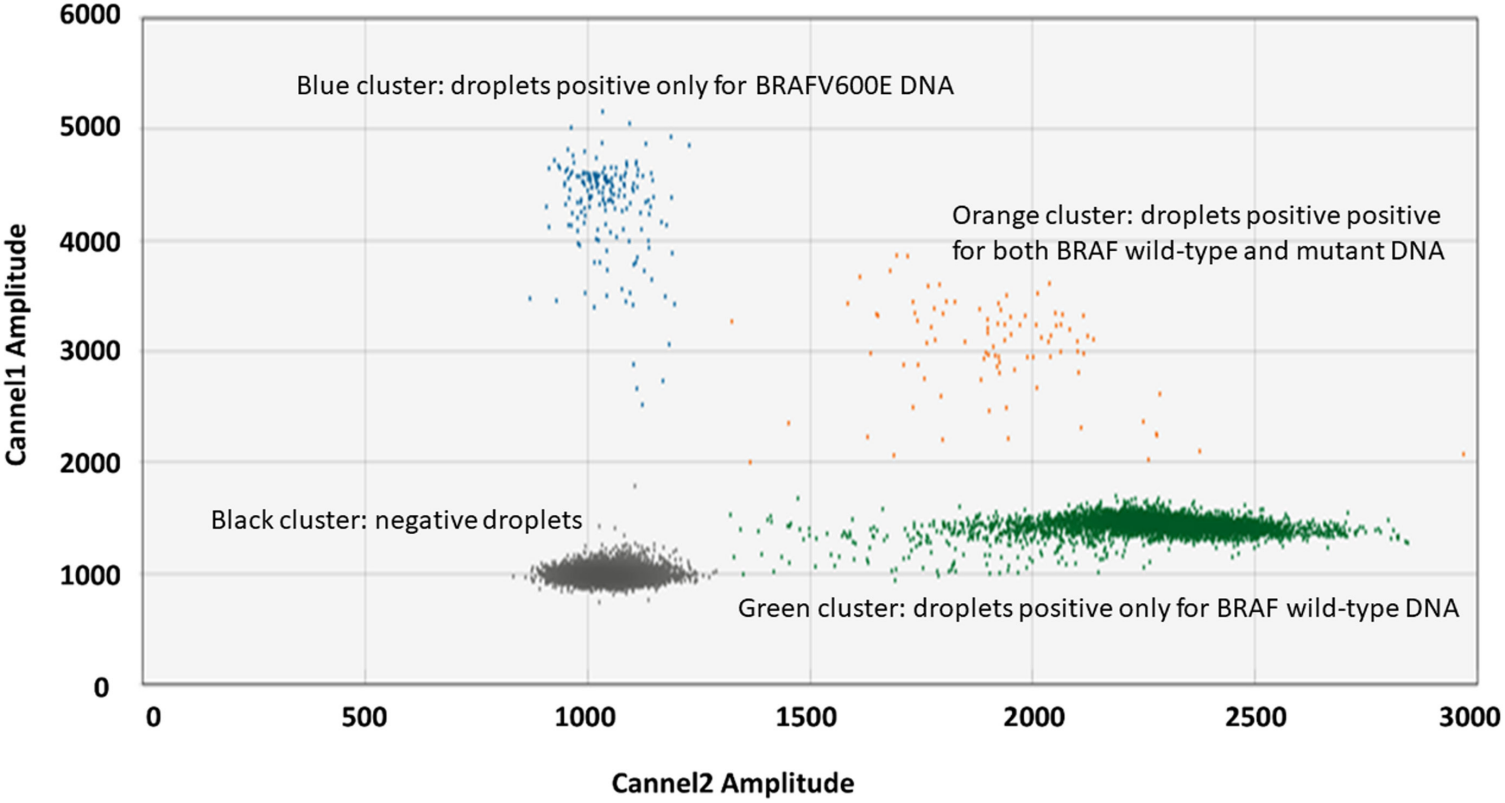
Example of BRAFV600E positive sample with 4.09 fractional abundance of mutant allele in formalin‐fixed paraffin‐embedded tissue melanoma tissue. Graph of two merged sample wells in ddPCR after preamplification. The green cluster represents droplets that are positive for BRAF wild‐type DNA only (HEX dye, channel 2). The blue cluster represents droplets that are positive for BRAFV600E mutant DNA only (FAM dye, channel 1). The orange cluster represents droplets that are positive for both wild‐type and mutant DNA (positive in both FAM and HEX channels).

Ultrasensitive digital droplet PCR (ddPCR)‐based method for the detection of BRAF mutations in plasma was used [18]. Q5 High‐Fidelity PCR Kit (New England BioLabs, MA) was used for the disproportionate pre‐amplification of mutant and wild‐type (WT) copies (favoring mutant alleles). After that DNA was purified using QIAquick PCR Purification kits (QIAGEN, Hilden, Germany). BRAFV600E mutations were detected in the pre‐amplified cfDNA using QX200 Droplet Digital PCR System (Bio‐Rad, CA) (Figure 1). Previous results from plasma BRAF testing have been reported. In this study, we have reported updated survival data in those patients as well as the results of a combined approach with plasma level of S100 [18].

### 2.5 Plasma Level of S100 Protein Assessment

S100 protein were quantitatively measured using automated sandwich immunoassay chemiluminescent method (CLIA) by kit LIAISON S100 (Ref.no. 314701), LIAISON S100 Cal (low/high) (Ref no. 319117) and LIAISON XL Analyzer (all DiaSorin S.p.A., Saluggia (VC), Italy). LIAISON Control S100 (Ref no. 319112) was used for quality control arrangement.

### 2.6 Data Processing and Statistical Analysis

Statistical analysis was done in the IBM SPSS Statistics for Windows, Version 26.0 (IBM Corp., NY). Median was used for description of continuous variables, while frequencies and percentages were used to describe categorical variables. Statistical significance was tested using Pearson's chi‐square test and Kruskal‐Wallis test. Survival analysis was done using Kaplan‐Meier analysis with log‐rank test to assess statistical significance. A *p* value of < 0.05 was considered statistically significant.

## 3 Results

Altogether 80 patients with melanoma of stages 0 to III with median age 60 years at diagnosis were included in the study (Table 1).

**TABLE 1:** Demographic information for the study participants.

Patient characteristics	Number of patients (%)
Participants in this study	80 (100%)
Gender	
Female	37 (46.3%)
Male	43 (53.7%)
Median age	60 years
Postoperative adjuvant therapy	22 (27.5%)
Interferon	16 (20%)
Nivolumab	5 (6.3%)
Dabrafenib + Trametinib	1 (1.3%)
Tumor stage	
0	4 (5%)
IA	12 (15%)
IB	12 (15%)
IIA	11 (13.7%)
IIB	10 (12.5%)
IIC	9 (11.2%)
IIIA	1 (1.3%)
IIIB	4 (5%)
IIIC	14 (17.5%)
IIID	1 (1.3%)
Unknown	2 (2.5%)
Relapse within follow‐up	
Yes	24 (30%)
No	56 (70%)
Death within follow‐up	
Yes	12 (15%)
No	68 (85%)

### 3.1 BRAF Mutations in Melanoma Tumor Tissue Samples

Melanoma tumor tissue samples for BRAF mutation analysis were available in 52 patients (65%). BRAF V600E mutation was detected in 23 of these 52 patients (44%). Tissue BRAF positivity was not predictive for patients' disease‐free interval (DFI; median not reached in both groups, *p* = 0.696), overall survival (OS; median not reached in both groups, *p* = 0.498), or probability of melanoma recurrence (34.6% vs. 38.5%, resp., *p* = 0.773), in comparison with tissue BRAF‐wild type (BRAF‐WT) melanoma.

### 3.2 BRAF Mutations in Preoperative and Postoperative Plasma Samples

Preoperative plasma samples for BRAF analysis were available in 76 patients (95%). BRAF V600E mutation was detected in 28 patients (36.8%). The mean preoperative plasma BRAF V600E fractional abundance (pFA; proportion of the mutant allele in total plasma DNA) was 0.23% ranging from minimum 0% up to maximum 6.6% and did not differ between disease stages (*p* = 0.316). The assessment of concordance between BRAF mutation status in tissue and preoperative plasma samples was possible in 49 patients. The overall percentage agreement between tissue and preoperative plasma BRAF mutation status (i.e., positive or negative) was 46.9% (23/49). In contrast, 26 patients (53.1%) experienced discordant status.

**FIGURE 2:**
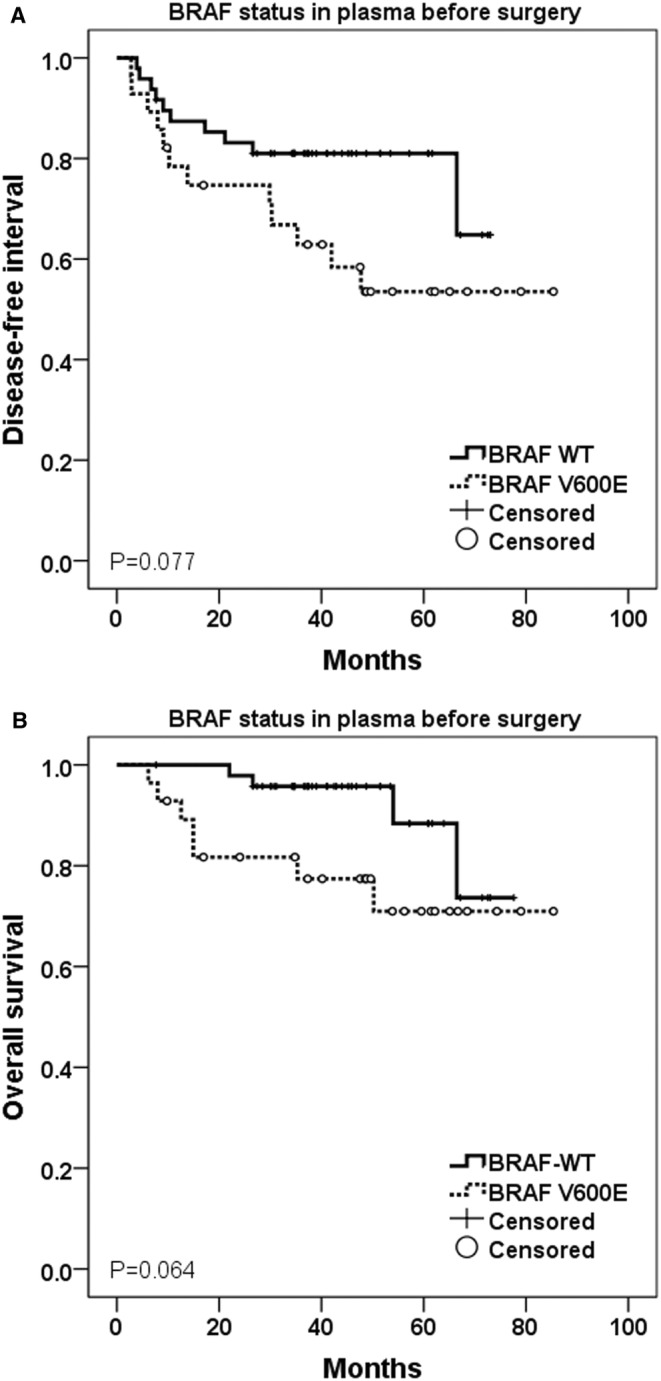
(A) Disease‐free interval (DFI) and (B) overall survival (OS) in association with BRAF mutation status (V600E mutation vs. wild‐type) in preoperative plasma samples (log‐rank test to assess statistical significance).

Patients with BRAF V600E mutation in preoperative plasma samples compared to BRAF‐WT experienced trend to worse DFI (median not reached in both groups, *p* = 0.077) as well as OS (median not reached in both groups, *p* = 0.064) (Figure 2A,B). Patients with BRAFV600E mutation in preoperative plasma samples had got also higher probability of melanoma recurrence after surgery (42.9% vs. 20.8%, resp., *p* = 0.041).

Early postoperative plasma samples for BRAF mutation analysis were available in 76 patients (95%). BRAF V600E mutation was found in 20 of them (26.3%). Conversion of BRAF V600E mutation into BRAF‐WT between the pre‐ and first postoperative plasma sample was observed in 15 patients (20%).

**FIGURE 3:**
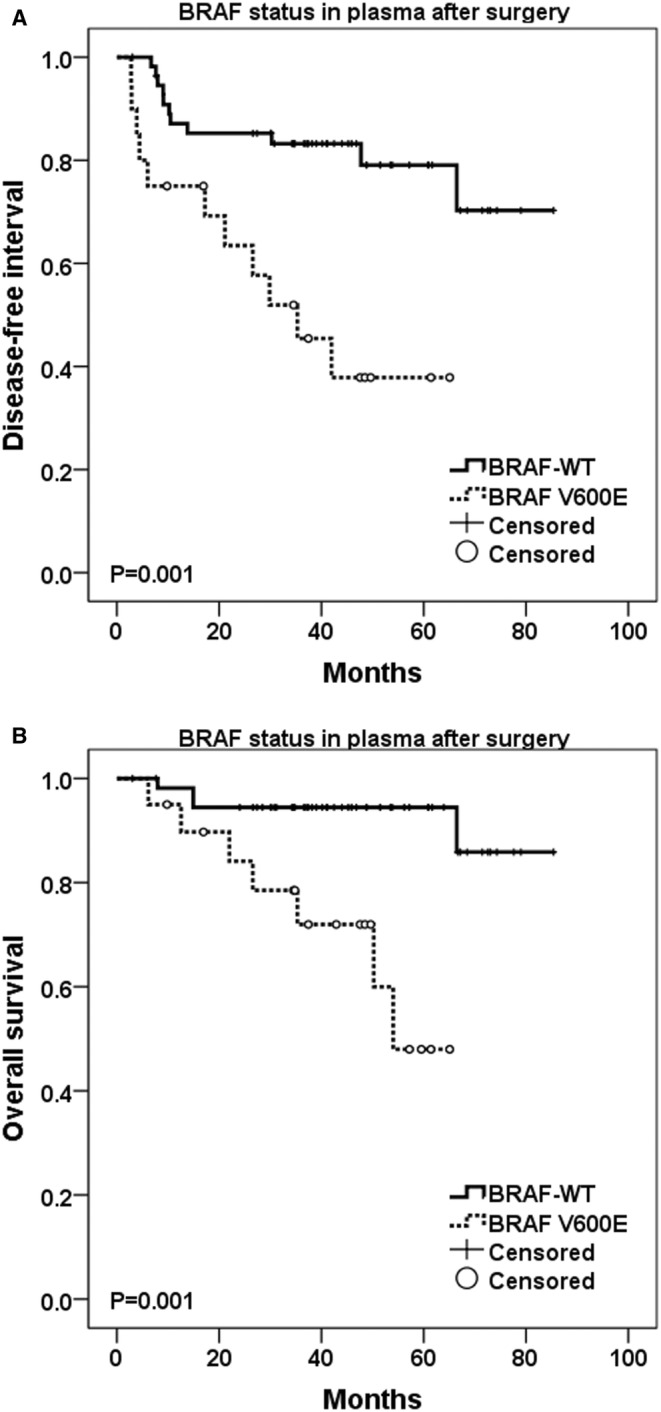
(A) Disease‐free interval (DFI) and (B) overall survival (OS) in association with BRAF mutation status (V600E mutation vs. wild‐type) in first postoperative plasma samples (log‐rank test to assess statistical significance).

In the survival analysis, patients with BRAF V600E mutation detected in first postoperative plasma sample experienced significantly shorter DFI (35.3 months vs. median not reached, *p* = 0.001), shorter OS (54 months vs. median not reached, *p* = 0.001), as well as higher probability of melanoma recurrence (55% vs. 19.6%, resp., *p* = 0.003) in comparison with BRAF‐WT (Figure 3A,B). Importantly, the presence of BRAF V600E mutation in first postoperative plasma sample was also independent predictor for worse OS (HR 7.746, *p* = 0.007) in multivariate analysis including other covariates (such as gender, age, disease stage, and application of adjuvant therapy), and there was a trend for worse DFI (HR 2.699, *p* = 0.062). On the other hand, percentage increment in postoperative BRAF V600E pFA was not associated with worse DFI (*p* = 0.326) or OS (*p* = 0.571).

The presence of BRAFV600E mutation in plasma samples obtained during the second postoperative time point also predicted higher probability of melanoma recurrence (47.8% vs. 24.1%, resp., *p* = 0.039) and shorter DFI (47.7 months vs. median not reached, *p* = 0.04), but not OS (*p* = 0.114). On the other hand, the plasmatic positivity of BRAFV600E mutation in any of the following post‐operative time points was not predictive for DFI, OS, or melanoma recurrence.

### 3.3 The Preoperative and Postoperative Plasma Levels of S100B Tumor Marker

**FIGURE 4:**
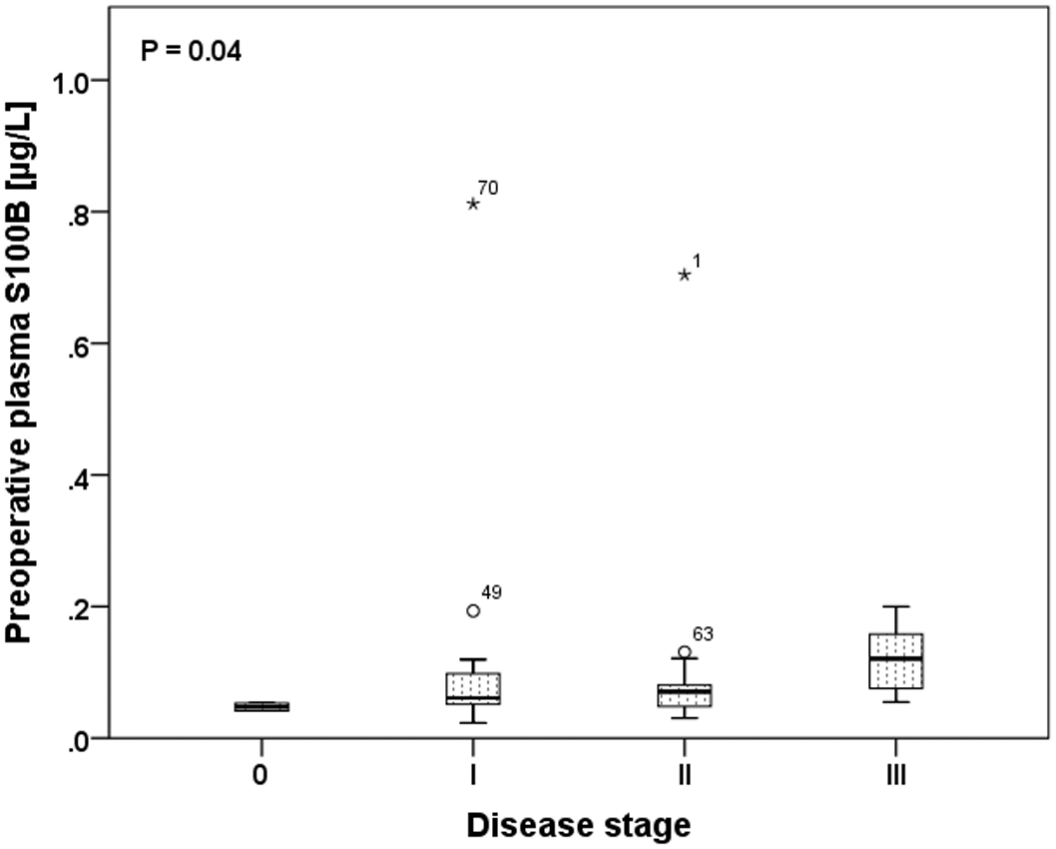
Preoperative plasma level of S100B tumor marker in accordance to disease stage (Kruskal‐Wallis test to assess statistical significance).

The preoperative plasma samples for the analysis of S100B tumor marker were available in 46 patients (57.5%). The mean preoperative S100B concentration was 0.112 μg/L ranging from minimum 0.0233 μg/L up to maximum 0.812 μg/L. Higher level of S100B in preoperative plasma sample compared to lower level (cut‐off value 0.0714 μg/L) was not predictive for patients’ DFI (*p* = 0.261), OS (*p* = 0.328), or melanoma recurrence (*p* = 0.332). However, S100B level increased with disease stages (Stage 0—median 0.0473 μg/L, Stage I—median 0.061 μg/L, Stage II—median 0.0708 μg/L, Stage III—median 0.1208 μg/L; *p* = 0.04; Figure 4).

The first postoperative plasma samples were available in 63 patients (78.8%) for S100B assessment. The mean postoperative S100B concentration was 0.231 μg/L ranging from minimum concentration 0.0399 μg/L up to maximum 2.149 μg/L. Similar to preoperative levels, higher level of S100B in postoperative plasma sample (cut‐off value 0.1466 μg/L) was not predictive for patients’ DFI (*p* = 0.573), OS (*p* = 0.208), or melanoma recurrence (*p* = 0.438). In contrast to preoperative level, the postoperative S100B concentration did not differ between disease stages (*p* = 0.293).

The higher level of S100B tumor marker was also not predictive for DFI or OS in plasma samples obtained during the second (*p* = 0.706 and *p* = 0.584, resp.), third (*p* = 0.221 and *p* = 0.483, resp.), and fourth (*p* = 0.859 and *p* = 0.554, resp.) postoperative time points.

### 3.4 Combined Detection of BRAF Mutations Together With S100B in Preoperative and Early Postoperative Plasma Samples

The combined detection of S100B and BRAF mutation status in preoperative and first postoperative plasma samples was available in 23 patients (28.8%) and 27 patients (33.8%), respectively. Those with both preoperative plasma BRAF V600E mutation and higher level of S100B experienced higher probability of melanoma recurrence (57.1% vs. 12.5%, resp., *p* = 0.025), shorter DFI (47.7 months vs. median not reached, *p* = 0.05), but not OS (median not reached in both groups, *p* = 0.131) compared to those with BRAF WT and lower S100B concentration (Table 2).

**TABLE 2:** Association of overall survival (OS) and disease‐free interval (DFI) with preoperative plasma levels of S100B tumor marker in combination with BRAF mutation status.

Preoperative plasma S100B level and BRAF mutation status			
	S100B high and BRAF V600E	S100B low and BRAF‐WT	*p*
DFI (months)	47.7	NR	*p* = 0.05
OS (months)	NR	NR	*p* = 0.131

**FIGURE 5:**
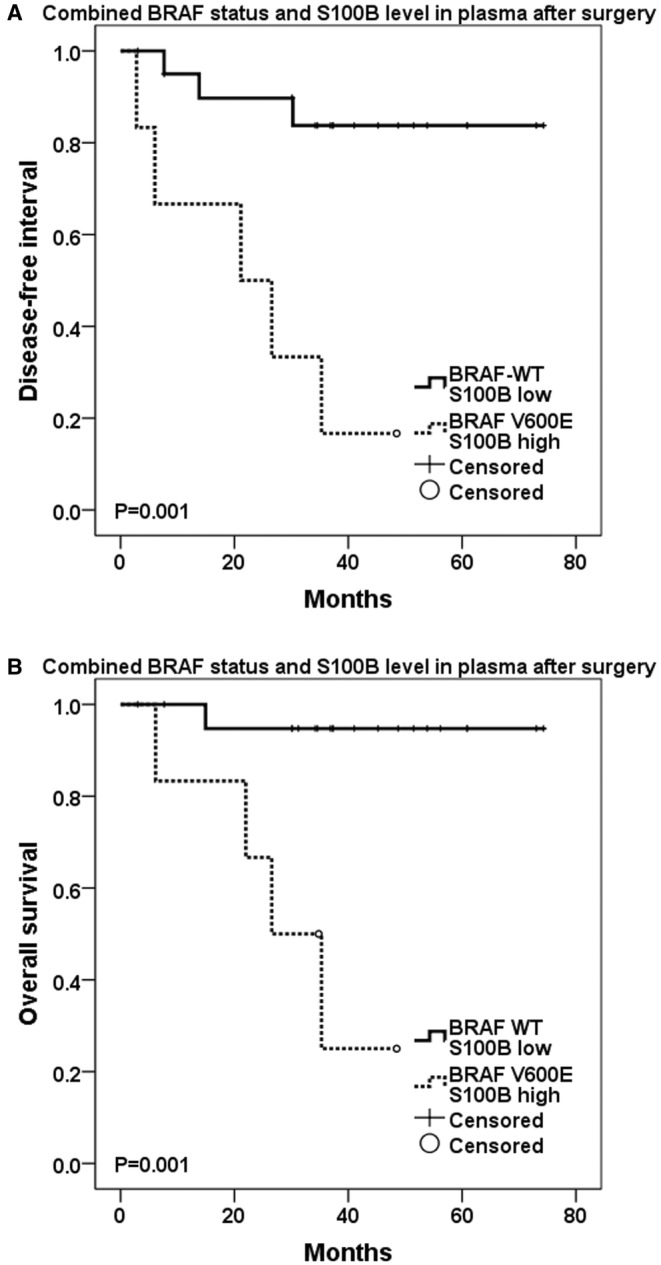
(A) Disease‐free interval (DFI) and (B) overall survival (OS) in association with postoperative levels of S100B tumor marker in combination with BRAF mutation status (log‐rank test to assess statistical significance).

Patients with BRAF V600E mutation and higher S100B level in first postoperative plasma sample experienced the highest probability of melanoma recurrence (83.3% vs. 14.3%, resp., *p* = 0.001) and the worst prognosis with the shortest DFI (21.1 months vs. median not reached, *p* = 0.001) and OS (26.6 months vs. median not reached, *p* = 0.001) compared to those with BRAF WT and lower S100B concentration (Figure 5A,B, Table 3). The combined postoperative presence of plasma BRAF V600E mutation and higher S100B level also best predicted shorter DFI (HR 19.76, *p* = 0.019) and OS (HR 54.761, *p* = 0.038) in multivariate analysis including other covariates (such as gender, age, disease stage, and application of adjuvant therapy).

**TABLE 3:** Association of overall survival (OS) and disease‐free interval (DFI) with first postoperative plasma levels of S100B tumor marker in combination with BRAF mutation status.

Postoperative plasma S100B level and BRAF mutation status			
	S100B high and BRAF V600E	S100B low and BRAF‐WT	*p*
DFI [months]	21.1	NR	*p* = 0.001
OS [months]	26.6	NR	*p* = 0.001

## 4 Discussion

Our study showed that the presence of mutated BRAF ctDNA and high levels of the tumor marker S100B in early postoperative blood samples predicted the highest probability of disease recurrence and the worst prognosis (Figure 5A,B). This combination has a higher predictive effect than discrimination based on the presence of the BRAF oncogene ctDNA only (postoperative: recurrence 83.3% vs. 55% of patients; preoperative: recurrence 57.1% vs. 42.9% of patients). Thus, the combination of low S100B levels and the absence of the ctDNA oncogene BRAF identifies patients who are most likely not to recur and adjuvant treatment would be less appropriate. This was also demonstrated using multivariate analysis by taking into account disease stage and other clinicopathological characteristics (age, gender, adjuvant therapy administration).

Although patients with early‐stage melanoma generally have a good prognosis, some of these patients may experience disease recurrence. Stage II patients have been found to have recurrence up to 44% of patients in the five‐year postoperative period [19]. Advances in the sensitivity of diagnostic methods using the liquid biopsy approach by analysis of circulating free tumor DNA (ctDNA) now allow the determination of relevant biomarkers in peripheral blood even in such low‐stage patients, opening up an effective possibility for their identification [20]. This goes hand in hand with the availability of modern adjuvant therapies based on targeted therapy with BRAF and MEK inhibitors or anti‐tumor immunotherapy using PD1 checkpoint inhibitors [21, 22, 23, 24].

The detection of ctDNA BRAF mutations in the blood appears to be an ideal marker for detecting patients at risk for several reasons. In general, higher levels of ctDNA molecules in the peripheral blood of patients with solid tumors are correlated with the progression of the primary disease, which is due to the intrinsic process of release of these molecules into the surrounding tumor and subsequent peripheral circulation [25]. In the case of melanoma, detection of ctDNA by determining the presence of mutations in the BRAF oncogene, which is the majority oncogene in this disease, is suggested. The administration of targeted therapy in the form of BRAF inhibitors is based on this fact [26]. At the same time, the determination of the presence of the BRAF oncogene in peripheral blood is a significant prognostic parameter [27]. For these reasons, it makes sense to use the determination of ctDNA of the BRAF oncogene in peripheral blood to identify high‐risk patients with early stages of melanoma using the most sensitive methods, such as droplet digital PCR.

Interesting study was published by Lee et al. [28] who demonstrated that preoperative ctDNA (BRAF, NRAS, and KIT oncogenes) predicted unfavorable prognosis. Similarly, Tan et al. [29] evaluated the prognostic value of detecting ctDNA levels (panel of BRAF, NRAS, TP53, KIT, and TERT mutations) in pre‐ and post‐operative plasma samples. Detection of those mutations in both pre‐ and postoperative samples was related to worse prognosis. Postoperative presence of ctDNA strongly predicted melanoma relapse. Both of these studies used ddPCR to detect the mutations in plasma and focused on stage III patients. As we mentioned, our study had similar results, but at the same time we can say that the above is valid for stage I‐III patients. Our research group has increased the sensitivity of this method by including a step based on unproportional preamplification [18]. By this approach, BRAF mutated ctDNA was observed in plasma samples collected before and after surgery in 36.8% and 26.3% of patients, resp.

However, it is important to keep in mind that mutations in the BRAF oncogene are not found in all melanoma patients, so we tested its combination with the conventional protein tumor biomarker S100B to identify high‐risk patients. Several studies have demonstrated the prognostic significance of the S100B tumor biomarker in generalized melanoma [30, 31, 32, 33]. Studies determining the S100B tumor marker in lower stages of melanoma have also been published, but these results are inconsistent in terms of their relationship to prognosis [34, 35]. In this study, we did not observe a statistically significant relationship of preoperative or postoperative S100B marker levels with disease prognosis or risk of recurrence. The plasma samples for this analysis were available in 46, resp. 63 patients. However, we did observe a significant increase in preoperative S100B levels in relation with disease stage. The meta‐analysis performed by Mocellin et al. [30] including 1594 patients with stage I‐III melanoma showed that elevated level of the tumor marker S100B is a negative prognostic factor for disease progression and survival (HR = 2.28, 95% CI: 1.8–2.89; *p* < 0.0001). This study demonstrates the benefit of determining S100B tumor marker levels in stages I‐III for determining prognosis. However, due to low specificity, there seems to be no consensus on the introduction of this marker into clinical practice in early stages. In our opinion, this limitation is overcome by the combination of this marker with the determination of molecular genetic markers at the DNA level, in our case the determination of the BRAF oncogene mutation in peripheral blood. The determination of ctDNA in the peripheral circulation, in addition to its high specificity and sensitivity in relation to the tumor, carries information about minimal residual disease after surgery.

The main limitation of this study is the number of patients enrolled into the study that subsequently results in the fact that the sub‐analysis of individual stages of the disease encounters a small number of patients. As our study was aimed to evaluate the prognosis and risk of recurrence in early‐stage melanoma, due to the long‐term follow‐up, the analysis included patients of the relevant stages who had not yet been treated with modern adjuvant therapy. Therefore, this is not a clearly predictive study with respect to adjuvant immunotherapy or targeted therapy.

## 5 Conclusions

We observed the benefit of the combination of protein tumor marker S100B and ctDNA BRAF oncogene in preoperative as well as postoperative peripheral blood samples for identification of patients with resectable melanoma (stage I–III) in high risk of recurrence and unfavorable prognosis. This assessment may help in clinical decision‐making process regarding the administration of modern adjuvant therapy in early‐stage melanoma patients.

## Author Contributions

**Jiri Polivka:** conceptualization (equal), investigation (equal), methodology (lead), writing – original draft (lead), writing – review and editing (lead). **Martin Pesta:** conceptualization (supporting), investigation (supporting), methodology (supporting), writing – original draft (supporting), writing – review and editing (supporting). **Mohamed A. Gouda:** conceptualization (equal), formal analysis (equal), investigation (equal), methodology (equal), writing – original draft (equal), writing – review and editing (equal). **Mahyar Sharif:** investigation (equal), methodology (supporting), writing – original draft (supporting). **Helen Huang:** investigation (equal), methodology (supporting). **Inka Treskova:** conceptualization (equal), data curation (equal), methodology (supporting), writing – review and editing (supporting). **Vlastimil Woznica:** data curation (equal), formal analysis (supporting), investigation (supporting). **Jindra Windrichova:** investigation (equal), methodology (supporting). **Katerina Houfkova:** investigation (equal), methodology (supporting). **Radek Kucera:** investigation (supporting), methodology (supporting), writing – review and editing (equal). **Tomas Fikrle:** data curation (supporting), investigation (supporting), writing – review and editing (supporting). **Jan Ricar:** writing – review and editing (supporting). **Kristyna Pivovarcikova:** investigation (supporting), methodology (supporting). **Ondrej Topolcan:** funding acquisition (equal), project administration (equal), supervision (equal). **Filip Janku:** conceptualization (equal), data curation (equal), formal analysis (equal), funding acquisition (equal), methodology (equal), project administration (equal), supervision (equal), writing – review and editing (equal).

## Conflicts of Interest

Filip Janku has research support from Astex, Novartis, BioMed Valley Discoveries, Fore Bio, Deciphera, Bristol‐Myers Squibb, Asana, Ideaya Biosciences, Sanofi, Merck, F‐star, JSI Innopharm, Bioxcel, Lilly, Bicara, PureTech Health, FujiFilm Pharmaceuticals, Sotio, Synlogic, NextCure, and Hutchinson Medipharma; is on the Scientific Advisory Boards of Ideaya Biosciences, Synlogic, Sotio, Puretech Health, Deciphera, Crown Bioscience, Asana, Fore Bio, Novartis, Bicara, and PegaOne; is a paid consultant for Mersana Therapeutics, Flame Bio, Cardiff Oncology, MedinCell, and Immunomet; has ownership interests in Cardiff Oncology and Monte Rosa Therapeutics; and has the leadership position in Monte Rosa Therapeutics. The remaining authors have declared no conflicts of interest.

## Data Availability

Data available on request from the authors.

## References

[1] L. E. Davis , S. C. Shalin , and A. J. Tackett , “Current State of Melanoma Diagnosis and Treatment,” Cancer Biology & Therapy 20, no. 11 (2019): 1366–1379. 31366280 10.1080/15384047.2019.1640032 PMC6804807

[2] L. A. von Schuckmann , M. C. B. Hughes , R. Ghiasvand , et al., “Risk of Melanoma Recurrence After Diagnosis of a High‐Risk Primary Tumor,” Journal of the American Medical Association Dermatology 155, no. 6 (2019): 688–693. 31042258 10.1001/jamadermatol.2019.0440 PMC6495363

[3] M. Saad , E. Castellano , and A. A. Tarhini , “Clinical Updates in Neoadjuvant Immunotherapy for Melanoma Before Surgery,” Expert Review of Clinical Immunology 1–17 (2023): 927–943. 10.1080/1744666X.2023.2248392 37578289

[4] M. S. Bagheri , J. Polivka , I. Treskova , et al., “Preoperative Plasma miRNA Levels Predict Prognosis in Early‐Stage Malignant Melanoma,” Anticancer Research 43, no. 2 (2023): 695–706. 36697090 10.21873/anticanres.16208

[5] L. Simetić , K. Blažičević , K. Međugorac , M. Golčić , and D. Herceg , “Relative Change in S100 as a Biomarker of Survival in Patients With Metastatic Melanoma Treated With Pembrolizumab,” Anticancer Research 40, no. 4 (2020): 2157–2163. 32234909 10.21873/anticanres.14175

[6] S. S. Ertekin , S. Podlipnik , S. Ribero , et al., “Monthly Changes in Serum Levels of S100B Protein as a Predictor of Metastasis Development in High‐Risk Melanoma Patients,” Journal of the European Academy of Dermatology and Venereology 34, no. 7 (2020): 1482–1488. 31967695 10.1111/jdv.16212

[7] E. A. Deckers , K. P. Wevers , A. C. Muller Kobold , et al., “S‐100B as an Extra Selection Tool for FDG PET/CT Scanning in Follow‐Up of AJCC Stage III Melanoma Patients,” Journal of Surgical Oncology 120, no. 6 (2019): 1031–1037. 31468535 10.1002/jso.25682 PMC6851671

[8] A. L. Frauchiger , R. Dummer , and J. Mangana , “Serum S100B Levels in Melanoma,” Methods in Molecular Biology 1929 (2019): 691–700. 30710305 10.1007/978-1-4939-9030-6_43

[9] C. Allgöwer , A.‐L. Kretz , S. von Karstedt , M. Wittau , D. Henne‐Bruns , and J. Lemke , “Friend or Foe: S100 Proteins in Cancer,” Cancers (Basel) 12, no. 8 (2020): 2037. 32722137 10.3390/cancers12082037 PMC7465620

[10] R. Harpio and R. Einarsson , “S100 Proteins as Cancer Biomarkers With Focus on S100B in Malignant Melanoma,” Clinical Biochemistry 37, no. 7 (2004): 512–518. 15234232 10.1016/j.clinbiochem.2004.05.012

[11] T.‐F. Xiong , F.‐Q. Pan , and D. Li , “Expression and Clinical Significance of S100 Family Genes in Patients With Melanoma,” Melanoma Research 29, no. 1 (2019): 23–29. 30216200 10.1097/CMR.0000000000000512 PMC6310472

[12] A. R. Bresnick , D. J. Weber , and D. B. Zimmer , “S100 Proteins in Cancer,” Nature Reviews. Cancer 15, no. 2 (2015): 96–109. 25614008 10.1038/nrc3893 PMC4369764

[13] E. Ricciardi , E. Giordani , G. Ziccheddu , et al., “Metastatic Melanoma: Liquid Biopsy as a New Precision Medicine Approach,” International Journal of Molecular Sciences 24, no. 4 (2023): 4014. 36835424 10.3390/ijms24044014 PMC9962821

[14] A. Tivey , F. Britton , J.‐A. Scott , D. Rothwell , P. Lorigan , and R. Lee , “Circulating Tumour DNA in Melanoma‐Clinic Ready?,” Current Oncology Reports 24, no. 3 (2022): 363–373. 35133615 10.1007/s11912-021-01151-6 PMC8885536

[15] P. Kamińska , K. Buszka , M. Zabel , M. Nowicki , C. Alix‐Panabières , and J. Budna‐Tukan , “Liquid Biopsy in Melanoma: Significance in Diagnostics, Prediction and Treatment Monitoring,” International Journal of Molecular Sciences 22, no. 18 (2021): 9714. 34575876 10.3390/ijms22189714 PMC8468624

[16] J. Polivka , M. Pesta , and F. Janku , “Testing for Oncogenic Molecular Aberrations in Cell‐Free DNA‐Based Liquid Biopsies in the Clinic: Are We There Yet?,” Expert Review of Molecular Diagnostics 15, no. 12 (2015): 1631–1644. 26559503 10.1586/14737159.2015.1110021 PMC5016094

[17] K. Pantel and M. R. Speicher , “The Biology of Circulating Tumor Cells,” Oncogene 35, no. 10 (2016): 1216–1224. 26050619 10.1038/onc.2015.192

[18] M. A. Gouda , J. Polivka , H. J. Huang , et al., “Ultrasensitive Detection of BRAF Mutations in Circulating Tumor DNA of Non‐metastatic Melanoma,” ESMO Open 7, no. 1 (2022): 100357. 34942440 10.1016/j.esmoop.2021.100357 PMC8695283

[19] F. C. Svedman , D. Pillas , A. Taylor , M. Kaur , R. Linder , and J. Hansson , “Stage‐Specific Survival and Recurrence in Patients With Cutaneous Malignant Melanoma in Europe – A Systematic Review of the Literature,” Clinical Epidemiology 8 (2016): 109–122. 27307765 10.2147/CLEP.S99021 PMC4887072

[20] M. Pesta , D. Shetti , V. Kulda , et al., “Applications of Liquid Biopsies in Non‐Small‐Cell Lung Cancer,” Diagnostics (Basel) 12, no. 8 (2022): 1799. 35892510 10.3390/diagnostics12081799 PMC9330570

[21] J. Larkin , M. Del Vecchio , M. Mandalá , et al., “Adjuvant Nivolumab Versus Ipilimumab in Resected Stage III/IV Melanoma: 5‐Year Efficacy and Biomarker Results From CheckMate 238,” Clinical Cancer Research 29, no. 17 (2023): 3352–3361. 37058595 10.1158/1078-0432.CCR-22-3145 PMC10472092

[22] A. M. M. Eggermont , C. U. Blank , M. Mandala , et al., “Longer Follow‐Up Confirms Recurrence‐Free Survival Benefit of Adjuvant Pembrolizumab in High‐Risk Stage III Melanoma: Updated Results From the EORTC 1325‐MG/KEYNOTE‐054 Trial,” Journal of Clinical Oncology 38, no. 33 (2020): 3925–3936. 32946353 10.1200/JCO.20.02110 PMC7676886

[23] R. Dummer , A. Hauschild , M. Santinami , et al., “Five‐Year Analysis of Adjuvant Dabrafenib Plus Trametinib in Stage III Melanoma,” New England Journal of Medicine 383, no. 12 (2020): 1139–1148. 32877599 10.1056/NEJMoa2005493

[24] J. J. Luke , P. Rutkowski , P. Queirolo , et al., “Pembrolizumab Versus Placebo as Adjuvant Therapy in Completely Resected Stage IIB or IIC Melanoma (KEYNOTE‐716): A Randomised, Double‐Blind, Phase 3 Trial,” Lancet 399, no. 10336 (2022): 1718–1729. 35367007 10.1016/S0140-6736(22)00562-1

[25] C. Bettegowda , M. Sausen , R. J. Leary , et al., “Detection of Circulating Tumor DNA in Early‐ and Late‐Stage Human Malignancies,” Science Translational Medicine 6, no. 224 (2014): 224ra24. 10.1126/scitranslmed.3007094 PMC4017867 24553385

[26] K. T. Flaherty , “A Twenty Year Perspective on Melanoma Therapy,” Pigment Cell & Melanoma Research 36, no. 6 (2023): 563–575. 37770281 10.1111/pcmr.13125

[27] Y. Zheng , H. Sun , L. Cong , et al., “Prognostic Value of ctDNA Mutation in Melanoma: A Meta‐Analysis,” Journal of Oncology 2021 (2021): 6660571. 34035810 10.1155/2021/6660571 PMC8116156

[28] J. H. Lee , R. P. Saw , J. F. Thompson , et al., “Pre‐Operative ctDNA Predicts Survival in High‐Risk Stage III Cutaneous Melanoma Patients,” Annals of Oncology 30, no. 5 (2019): 815–822. 30860590 10.1093/annonc/mdz075 PMC6551453

[29] L. Tan , S. Sandhu , R. J. Lee , et al., “Prediction and Monitoring of Relapse in Stage III Melanoma Using Circulating Tumor DNA,” Annals of Oncology 30, no. 5 (2019): 804–814. 30838379 10.1093/annonc/mdz048 PMC6551451

[30] S. Mocellin , G. Zavagno , and D. Nitti , “The Prognostic Value of Serum S100B in Patients With Cutaneous Melanoma: A Meta‐Analysis,” International Journal of Cancer 123, no. 10 (2008): 2370–2376. 18752249 10.1002/ijc.23794

[31] E. A. Janka , T. Várvölgyi , Z. Sipos , et al., “Predictive Performance of Serum S100B Versus LDH in Melanoma Patients: A Systematic Review and Meta‐Analysis,” Frontiers in Oncology 11 (2021): 772165. 34950582 10.3389/fonc.2021.772165 PMC8688362

[32] N. B. Wagner , A. Forschner , U. Leiter , C. Garbe , and T. K. Eigentler , “S100B and LDH as Early Prognostic Markers for Response and Overall Survival in Melanoma Patients Treated With Anti‐PD‐1 or Combined Anti‐PD‐1 Plus Anti‐CTLA‐4 Antibodies,” British Journal of Cancer 119, no. 3 (2018): 339–346. 29950611 10.1038/s41416-018-0167-x PMC6070917

[33] A. Karonidis , M. Mantzourani , H. Gogas , and D. Tsoutsos , “Serum S100B Levels Correlate With Stage, N Status, Mitotic Rate and Disease Outcome in Melanoma Patients Independent to LDH,” Journal of the Balkan Union of Oncology 22, no. 5 (2017): 1296–1302. 29135116

[34] G. Dumitraşcu , C. Constantin , G. Manda , et al., “Serum Markers in Skin Melanoma—Preliminary Study,” Roumanian Archives of Microbiology and Immunology 68, no. 3 (2009): 125–135. 20361532

[35] L. Lugović , M. Situm , M. Buljan , S. Poduje , and K. Sebetić , “Results of the Determination of Serum Markers in Patients With Malignant Melanoma,” Collegium Antropologicum 31, no. Suppl 1 (2007): 7–11. 17469741

